# The Battle to Sequence the Bread Wheat Genome: A Tale of the *Three Kingdoms*

**DOI:** 10.1016/j.gpb.2019.09.005

**Published:** 2020-06-17

**Authors:** Jiantao Guan, Diego F. Garcia, Yun Zhou, Rudi Appels, Aili Li, Long Mao

**Affiliations:** 1 National Key Facility for Crop Gene Resources and Genetic Improvement, Institute of Crop Science, Chinese Academy of Agricultural Sciences, Beijing 100081, China; 2 Collaborative Innovation Center of Crop Stress Biology & Institute of Plant Stress Biology, School of Life Science, Henan University, Kaifeng 475004, China; 3 AgriBio, Centre for AgriBioscience, Department of Economic Development, Jobs, Transport, and Resources, La Trobe University, Melbourne, VIC 3083, Australia

**Keywords:** Common wheat, Homoeologous genomes, Polyploid, Sequencing, *Triticum aestivum*

## Abstract

In the year 2018, the world witnessed the finale of the race to sequence the genome of the world’s most widely grown crop, the **common wheat**. Wheat has been known to bear a notoriously large and complicated genome of a polyploidy nature. A decade competition to sequence the wheat genome initiated with a single consortium of multiple countries, taking a conventional strategy similar to that for **sequencing** Arabidopsis and rice, became ferocious over time as both sequencing technologies and genome assembling methodologies advanced. At different stages, multiple versions of genome sequences of the same variety (*e.g.*, Chinese Spring) were produced by several groups with their special strategies. Finally, 16 years after the rice genome was finished and 9 years after that of maize, the wheat research community now possesses its own reference genome. Armed with these genomics tools, wheat will reestablish itself as a model for **polyploid** plants in studying the mechanisms of polyploidy evolution, domestication, genetic and epigenetic regulation of homoeolog expression, as well as defining its genetic diversity and breeding on the genome level. The enhanced resolution of the wheat genome should also help accelerate development of wheat cultivars that are more tolerant to biotic and/or abiotic stresses with better quality and higher yield.

## *Prelude*

*The popular classical Chinese novel The Romance of Three Kingdoms tells the story of the three Kingdoms – Wei, Shu, and Wu, which co-existed after numerous land-scrambling battles 1700 years ago. The race to conquer the genome of wheat, named Chinese Spring, with the three genomes A, B, and D reminds one of this historical period. The wheat genome was competitively sequenced multiple times by several groups. The game ended with a high quality Chinese Spring genome sequence produced by the International Wheat Genome Sequencing Consortium (IWGSC) in August 2018. The race to complete the long-awaited genome of bread wheat, after 16 years of the completion of the rice genome and 9 years of that of maize, is legendary and worthy to retrospect at this exciting moment and to look forward for a new dynasty of wheat research and breeding.*

## Introduction

Common wheat (*Triticum aestivum* L.) is the most widely grown crop on Earth. Bread contributes 20% of calories that humans need and is one of the most important protein resources [1]. The genome of wheat however is extremely complicated relative to those of other crops, because it is composed of three subgenomes A, B, and D. Current knowledge considers that common wheat was derived from a hybridization between cultivated tetraploid wheat (*T. turgidum*, ssp *dicoccum*, AABB, 2 N = 4 × = 28) and the goat grass *Aegilops tauschii* (DD, 2 N = 2 × = 14) [2]. The A genome donor is considered to be *T. urartu* (AA), while the source of the B genome is not clear [3]. The hexaploid nature of the wheat genome however is pivotal to making common wheat a better crop than its progenitors, because interactions between subgenomes contribute to its flexibility in gene expression levels, which may underlie its enhanced adaptability to various environments [4].

For a long time, wheat was a good model for cytogenetics and genetics studies because of dosage compensation between homoeologous genes and chromosomes. The landrace Chinese Spring (CS), originally from Chengdu, Sichuan in China, played an important role by providing a large number of genetic stocks developed by E. R. Sears, including a whole set of chromosome additional lines, deletion lines, and nulli-tetrasomic lines that allowed dissection of chromosome behavior and genetic loci that underlie important agronomic traits [5], [6], [7]. Thus, wheat contributed significantly to our understanding of plant chromosomes and their inheritance.

## The dark age of wheat genome research

In the year 2000, a genome sequencing strategy based on the bacterial artificial chromosome (BAC) physical maps and conventional ABI737 sequencing machines was used for the human genome sequencing with a price tag of several billions of dollars [8], [9]. In the same year, the first plant genome, the Arabidopsis genome, was sequenced using this strategy [10]. Despite the low throughput of the equipment, the reads were long. Two years later, multiple versions of rice genomes were completed [11], [12]. Since then, Arabidopsis and rice with their enviously small genomes, 125 and 466 megabases (Mb), respectively, have been hailed as models for plant genetics and genomics.

The hexaploid nature of wheat makes its genome of 16,000 Mb, nearly 40 times that of rice. This made it economically unaffordable for a standard sequencing approach. To make things even more difficult, the wheat genome contains a high percentage of repetitive sequences and homoeologous DNA copies from the three subgenomes, both of which pose severe challenges for proper assembly of the genome. Under such circumstances, an international collaborative effort was needed. In 2005, the International Wheat Genome Sequencing Consortium (IWGSC) was initiated, dividing the gigantic job on the basis of chromosomes and chromosome arms among 20 participating countries. This strategy utilized the genetic stocks that were able to distinguish individual chromosomes and separable by flow cytometry [13]. BAC libraries were generated and fingerprinted to produce physical maps and minimum tiling paths that were then sequenced and assembled [14]. Even this chromosome by chromosome method took almost a decade to establish and was only partially achieved for a few chromosomes such as chromosome 3B [15], where the initial motive was to assist cloning of agronomically important genes such as the *Fusarium* head blight gene on the short arm of chromosome 3B [16]. The size of hexaploid wheat genome also compelled some researchers to take an alternative course of targeting the genomes of related diploid species such as *Ae. tauschii*, with a genome a third of that of hexaploid wheat (~4.792 gigabases or Gb) and no interference from homoeologous DNA copies for physical mapping and eventual sequence assembly. Even with this approach, the task was daunting using regular agarose gels at first, then engaging higher throughput technologies such as the SNaPshot BAC fingerprinting technology and the Illumina Infinium SNP array technology for contig anchoring. It took 10 years to get the first version of the *Ae. tauschii* physical map, where a total of 461,706 BAC clones were fingerprinted and assembled into 2263 contigs that were anchored to a genetic map using 7185 molecular markers [17]. The map was estimated to contain 4.03 Gb of genomic sequences, covering 84% of the genome.

This was a dark age for wheat genomics research. Only a handful of genes were cloned in wheat [18], [19], [20], [21] while watching the steady progress in genome sequencing of additional two brother species, sorghum and maize, that were completed in 2009 [19], [22], [23]. In rice, functional genomics was carried out like a raging fire where a number of agronomically important genes were cloned and published [24], [25], [26], [27], [28] and thus had a much greater impact on both plant biology and plant biologists. In contrast, wheat biology research was severely hampered and seminars were rarely heard in major plant conferences. A quality wheat genome was eagerly expected, but was nowhere in sight.

## The dawning of wheat genome sequencing

By 2010, the Illumina HiSeq 2000 machine was able to perform sequencing for paired-end reads of 100 bp long, with a throughput of 25 Gb per day and 200 Gb sequence per run, providing hopes to reach low copy regions of complex genomes like the wheat genome using a shotgun sequencing strategy.

Two diploid progenitors of common wheat were first to be decoded. The draft genome sequence of the D genome progenitor *Ae. tauschii* was achieved using an approximately 90× genome coverage of short reads from libraries with various insert sizes [29]. The wheat variety used was AL8/78. SOAPdenovo (version 1.05) [30] was used to assemble the filtered short reads (<100 bp) achieving contigs with an N50 of 4.51 kb. The size of assembled scaffolds equaled 83.4% of the genome and 65.9% of them were annotated as transposable elements (TEs). Despite the short contig size, a total of 43,150 protein-coding genes were identified with the assistance of RNA-seq data of various tissues. A high-density genetic map of 151,083 markers was developed to anchor 30,697 (71.1%) genes to chromosomes. Consistent with extant observations, gene families for disease resistance, abiotic stress tolerance, and grain quality were expanded in the genome of *Ae. tauschii*, providing an explanation at the molecular level for the role of *Ae. tauschii* to enhance the adaptation of hexaploid wheat. The genome sequence of the A genome donor, *T. urartu* (accession G1812), was developed using a similar strategy [31]. *T. urartu* is one of the domesticated types of diploid wheat (AA, 2 N = 2 × = 28). In total, 448.49 Gb of short reads from the Illumina HiSeq 2000 platform were generated from short insert size libraries of 200, 350, 500, and 700 bp and were assembled using SOAPdenovo (v. 1.05). The resulting contigs totaled 3.92 Gb in length, representing 79.35% of the estimated A genome (4.94 Gb) with an N50 of merely 3.42 kb. Gene model prediction, genome structure comparison, and assessment of its utility for agronomically important gene discovery and for developing molecular markers were performed.

The genomes of progenitors cannot completely represent their counterparts in the common wheat genome because of interactions between subgenomes and evolution during the approximately 10,000-year period since the hexaploid genome came into being. Therefore, common wheat (variety CS) remained as an important target for establishing its own genome sequence. The first common wheat genome sequence was obtained by a different sequencing platform, the Roche 454 pyrosequencing machine (GS FLX Titanium and GS FLX1 platforms), which was able to generate reads up to 500 bp with a throughput of 400 Mb per run. A total of 22 million shotgun reads (85 Gb) were generated and corresponded to around 5× depth of the estimated hexaploid wheat genome (16 Gb). To compensate for the low coverage by Roche 454 sequencing and for the genome assembling, additional sequences from various platforms and related progenitors were produced, such as Illumina reads from *T. monococcum*, another diploid wheat with an A genome, the 454 sequences of *Ae. tauschii*, and cDNA sequences from *Ae. speltoides* that confers a genome comparable to the B genome. Additional CS short reads were also produced using the SOLiD platform. Nonetheless, genome assembling was a challenging task. First, 454 sequences were clustered with orthologous grass gene sequences and assembled separately under a high stringency of Newbler, a commercial software developed by Roche. Second, repetitive sequences were filtered out and the remaining low copy number sequences were assembled. Third, cDNA sequences from multiple resources were assembled to help gene annotation using MetaSim [32]. Finally, a total of 95,000 gene models were predicted with two thirds of them being assigned to the three subgenomes. Although the draft genome was highly fragmented, it was the first wheat genome that the community could work with [33].

Meanwhile, the progress of the chromosome-based BAC by BAC sequencing adopted by the IWGSC went along steadily. Sequencing libraries were developed from the DNA of individual chromosomes or chromosomes arms and were sequenced on the Illumina HiSeq 2000 platform with the pair-end mode. Since the reads were still very short, *de novo* ABySS designed for short reads assembling [34] was applied. The resulting 10.2 Gb assembly, similar to the 454 assembly, contained nearly half million contigs with the N50 ranging 1.7–8.9 kb. Together these contigs represent 61% of the estimated hexaploid wheat genome. A total of 133,090 so called high confidence (HC) genes were predicted, with 890,576 low confidence (LC) genes containing ORF-like structures. Assisted with a genetic map, a little over half (56%) of the HC genes were anchored genetically [35] and could subsequently be considered in the context of the 42 telosome-based sequence resource of short assemblies for each chromosome arm. As a result, a draft genome of wheat referred to as the IWGSC chromosome survey sequence (CSS) assembly (IWGSC 2014) was provided, which by then was the best genome for wheat researchers.

An outstanding achievement accomplished by the IWGSC at the time was the production of a reference level sequence of chromosome 3B [36]. This high-quality chromosome sequence was produced from a minimum tiling path of 8452 BACs and was 774 Mb long, carrying 5326 protein-coding genes and 85% of TEs, combined with a detailed molecular-genetic map (CS × Renan) for long-range orientation of DNA sequences. The assembly of chromosome 3B was a snapshot for the remaining 20 projects and was a proof of concept that this chromosome-based BAC by BAC strategy could succeed, given sufficient time.

## Acceleration of wheat genome sequencing

The development of sequencing technologies in both throughput and read length such as the long Pacific Biosciences (PacBio) reads, combined with libraries of various insert sizes and other physical measurement techniques such as Hi-C, an extension of chromosome conformation capture (3C) [37], allowed development of novel algorithms and pipelines to assemble complex genomes such as that of wheat in a much faster and accurate fashion.

The first test drive using a new hybrid assembly technique that combined the PacBio reads, which are long but error-prone, and Illumina reads, which are short but accurate, was performed using sequences of *Ae. tauschii* [38]. The strategy involved more than 19 million PacBio reads (~38 × D genome coverage), 177 × Illumina HiSeq 2500 reads (200-bp paired-end reads) and MiSeq reads (250-bp paired-end reads), reaching a 200 × genome coverage of sequences from libraries of various insert sizes. MaSuRCA, a newly developed pipeline that can accommodate both long and short reads, was applied, resulting in an assembly Aet_MR.1.0 that contained 128,898 scaffolds with a total length of 4.778 Gb. Although there were still a lot of contigs with no genome annotation, both contig size and scaffold length were significantly improved with N50s reaching 486.8 kb and 521.7 kb, respectively. Importantly, the quality of the genome was proved by comparing with independently produced optical maps and a set of high-quality BAC-based assemblies.

The pipeline was then used to generate the first near-complete hexaploid wheat (CS42) assembly that was not oriented against a molecular-genetic map [39]. First, a total of 100 × genome coverage sequences were produced: 65 × were from 7.06 billion Illumina 150-bp paired-end reads and the other 36 × were from 55.5 million PacBio reads. MaSuRCA was applied to generate 95.7 million super-reads, which were used to generate 57,020,767 mega-reads by aligning them to the PacBio reads (an N50 of 8.427 kb). Additional synthetic mate pairs were created to link mega-reads and together were assembled by the Celera Assembler (v8.3) [40]. This genome version was called Triticum 1.0 and was composed of 829,839 contigs, totaling 17.05 Gb with a contig N50 of 76.3 kb and the scaffold N50 of 101.2 kb. A second approach was to assemble the long reads directly using the FALCON assembler, resulting in FALCON Trit1.0 of 12.94 Gb. This version was shorter than the one assembled by MaSuRCA, but had a longer contig N50 (215.3 kb) among 97,809 contigs. The merging of Triticum 1.0 and Trit1.0 using MUMmer [41] generated a final assembly of 15.3 Gb with a contig N50 of 232.6 kb, covering nearly the complete wheat genome.

The second effort to assemble the CS genome using a more direct method was conducted by using optimized data types and specially designed algorithms [42]. In this work, 1.1 billion 250-bp paired-end reads were produced (33 × genome coverage) from the CS short insert libraries. For scaffolding, libraries of 2.5–11.6 kb inserts that provided long mate-pair (LMP) were developed and sequenced to 53 × genome coverage, with additional 15 × sequence coverage reads from so-called Tight, Amplification-free, Large insert pair-end Libraries (TALL). The assembly program w2rap-contigger generated almost 3 million contigs (the minimum length >500 bp) with an N50 of 16.7 kb [43]. SOAPdenovo was used for scaffolding [44], reducing the number of contiguous sequences to 1.3 million with an N50 of 83.9 kb. CSS-survey reads were used to anchor scaffolds to chromosomes. This version of the wheat genome assembly, called TGACv1, was 13.43 Gb long and represented more than 78% of the wheat genome. Besides the improved assembly, this version was also better annotated by combining strand-specific Illumina RNA-seq and PacBio full-length cDNAs. Although none of the two assemblies mentioned above reached the chromosome level, a decent portion of the wheat genome was now available for the community to use and a quality reference genome was almost in sight.

## The peak time of wheat genome sequencing

The years 2017–2018 marked a milestone in wheat genome sequencing. Solutions to sequencing complex genomes appeared to be routine and a number of genomes for wheat of different polyploidy levels were decoded. The first reference level genome of polyploid wheat, the genome of wild emmer wheat (WEW), was sequenced, assembled, and published in the time-frame of only a few years using the software developed by a young company called NRGene [45]. The genome was accomplished by obtaining a total of 176 × genome coverage reads from five libraries with insert sizes ranging from 450 bp to 10 kb, totaling 2.1 Tb that were generated on Illumina HiSeq 2500 sequencing machines. The newly developed software DeNovoMAGIC2 was used for contig and scaffold construction. Scaffolds were then integrated using a high-density molecular-genetic linkage map and further assembled using reads from the 3-dimensional (3D) HiC library. The final assembly was 10.5 Gb, representing 87.5% of the estimated tetraploid wheat genome. This chromosome scale reference genome had a contig N50 of 57 kb and a scaffold N50 of nearly 7 Mb. The high quality of this genome sequence was supported by the annotation of 110,544 gene models. Among these, 58.8% (65,012) were HC gene models and 41.2% were LC ones. As validated by BUSCO [46], this assembly captured 98.4% of the total expected gene sets of WEW. The assembly has already shown its usefulness by identifying domestication genes in this tetraploid species, such as genes responsible for grain threshing that have been puzzling wheat researchers for years [45]. Although published in 2019, the long undertaking genome sequencing of durum wheat (DW), which produces pasta, was finished with a similar assembling strategy as its wild counterpart, WEW, using the software package DenovoMAGIC2 of NRGene [47]. Assisted with a survey of 1856 Global Tetraploid Wheat Collection accessions, the genetic diversity and selection signatures could be associated with domestication and breeding. More importantly, this pasta producing species was found to carry a mutated allele of a metal transporter gene that appeared during domestication and rendered increased accumulation of high cadmium in grains. The beneficial allele in the wild species was identified as useful for improving wheat cultivars that are widely used by consumers today.

The WEW and DW genome assemblies provided good examples for completing a complex genome in a relative short time and in a more straightforward manner. The DeNovoMAGIC2 program was used at various steps by the two *Ae. tauschii* genome sequencing projects. Luo et al assembled reads from BACs using SOAPdenovo2 and those from the whole genome shotgun sequencing using DeNovoMAGIC2 [48]. The two assemblies were then merged using the MaSuRCA assembler [49], with additional PacBio sequencing data to close the gaps between scaffolds. The version Aet v4.0 has a combined length of the pseudomolecules of 4.02 Gb that were produced according to three different optical BioNano genome (BNG) maps and one linkage map, representing 95.2% sequences in the scaffolds [48]. A similar approach was adopted by Zhao et al to increase both contig and scaffold N50s [50]. Again, DeNovoMAGIC2 was used to assemble reads from both HiSeq 2000 and HiSeq 2500 from libraries of 450-bp and 8-kb sequencing libraries, which gave a contig N50 of 50.3 kb and a scaffold N50 of 6.8 Mb. The contig N50 was increased to 112.6 kb using the long reads from the PacBio platform, while SSPACE [51] was used to increase the scaffold N50 to 13.1 Mb by using Illumina short reads from 20-kb and 40-kb libraries [50]. This version of the *Ae. tauschii* reference sequence anchored 4.32 Gb sequences to the pseudo-molecules, an increase of nearly 200 fold in sequence continuity compared to its first version [29]. To update the *T. urartu* genome, Ling et al adopted a similar strategy to Luo et al where physical map information was consulted during the sequence assembling process [48], [52]. MaSuRCA, instead of DeNovoMAGIC2, was used to assemble high-coverage shotgun reads of BAC clones while PacBio SMRT reads and linked reads were used with optical mapping for scaffolding. Using a genetic linkage map, the scaffolds were allocated into seven pseudomolecules totaling 4.79 Gb with a contig N50 of 344 kb and a scaffold N50 of 3.67 Mb, representing 97% of the estimated *T. urartu* genome (4.94 Gb) [52]. Judging from their contig and scaffold N50s, the genomes of these common wheat progenitors all reached a high quality reference sequence level.

**Figure 1:**
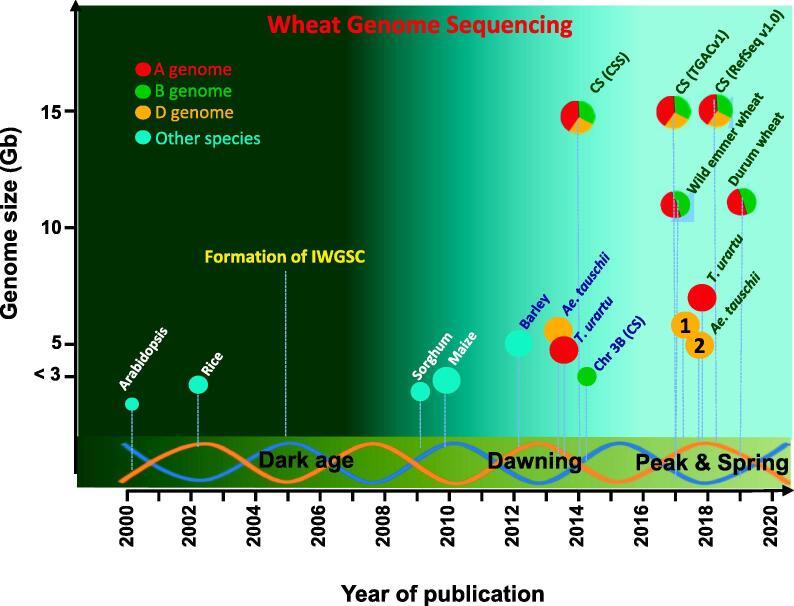
**A chart depicting the progress of wheat genome sequencing** The International Wheat Genome Sequencing Consortium (IWGSC) was formed in 2005. IWGSC agreed to use the bread wheat (*Triticum aestivum* L.) variety Chinese Spring (CS) as a consensus accession to build a reference wheat genome due to numerous genetic stocks available for this variety. *T. urartu* is the donor of the wheat A subgenome (AA, 2 N = 2 × = 14), while *Ae. tauschii* is the donor of the wheat D subgenome (DD, 2 N = 2 × = 14). First draft assemblies of *Ae. tauschii* and *T. urartu* were reported in 2013 [29], [31]. Two reference quality assemblies of *Ae. tauschii*, 1 and 2, were published in 2017 [48], [50], while the reference quality assembly of *T. urartu* was reported in 2018 [52]. The domesticated form of wild emmer wheat (WEW), *T. turgidum* (AABB, 2 N = 4 × = 28), is the putative tetraploid donor of common wheat, and durum wheat (DW) is a cultivated emmer wheat to make spaghetti (AABB, 2 N = 4 × = 28). The two tetraploid wheat WEW and DW were sequenced in 2017 and 2019, respectively [45], [47]. CS (CSS) is a draft CS genome sequence assembed with various short chromosome survey sequences available in 2014 [13], together with the first continous chromosome, the chromosome 3B (Chr 3B) of CS [36]. CS (TAGCv1) is an improved CS genome assembly with larger and more continuous scaffolds and better annotations published in 2017 [42], while CS (RefSeq v1.0) is the first reference quality genome assembly published in 2018 [53], which has been continuously improved since then (http://www.wheatgenome.org/).

With all needed tools at hand, the fruit of hexaploid common wheat reference genomes was ripe to pick. In August 2018, a high quality reference genome was released, 16 years after the rice genome and 9 years after the maize genome! The production of this IWGSC RefSeq v1.0 was achieved using DeNovoMAGIC2 assembled whole genome frame as a backbone, with integration of previously generated physical maps, genotyping-by-sequencing data, radiation hybrid maps, BioNano optical maps, and Hi-C data [53]. Twenty-one chromosome-scale pseudomolecules were allocated to the three subgenomes A, B, and D. The 14.5 Gb genome assembly conferred contigs and scaffolds with N50s as 52 kb and 7 Mb, respectively, while the super-scaffold N50 even reached 22.8 Mb! The long-awaited common wheat genome had finally arrived (Figure 1).

## The spring of wheat functional genomic research

The availability of the high quality reference genomes of wheat and its progenitors allows many questions to be asked that have been puzzling wheat researchers for a long time. The common wheat genome immediately provides the first glimpse of how the A, B, and D subgenomes evolve once they formed a hexaploid genome.

First of all, the genome sequences indicate that the composition of the three subgenomes is largely similar [53]. The numbers of HC protein-coding genes were nearly equal on the three subgenomes (35,345, 35,643, and 34,212 for the A, B, and D subgenomes, respectively) (Table 1). In addition, the content of TEs (up to 85% of each subgenome) was relatively equal across the three subgenomes as well (Table 2). Such patterns were also observed between A and B subgenomes in tetraploid emmer wheat [45]. Despite these, pseudogenes were obviously fewer on the D subgenome (81,905) compared with those on A and B (99,754 and 109,097 respectively) in hexaploid wheat. Differences were also observed between the genomes of diploid progenitors and their counterparts in polyploid wheat. As shown in Table 1, the *T. urartu* genome was predicted to confer 41,505 HC genes, 10,775 more than the A subgenome of WEW and 5203 more than in the A subgenome in CS (Table 1). On the other hand, the *Ae. tauschii* genome confers 5501 more genes than the CS D subgenome, although the B subgenome of WEW has 4655 fewer genes than the B subgenome of CS. The discrepancy between the subgenomes and genomes of wheat progenitors is in contrast to the similarity in gene numbers between subgenomes in polyploid wheat, suggesting somewhat convergent evolution of these subgenomes. Since all these genomes are in high quality, such comparison should reliably reflect the genome evolution rates in polyploid wheat and their diploid progenitors.

**Table 1:** The statistics of wheat reference genome assemblies

**Table 2:** Percentages of transposable elements in various wheat reference genomes

*Note*: SINE, short interspersed nuclear element; LINE, long interspersed nuclear element; MITE, miniature inverted transposable element.

A sequenced genome is essential to study the landscape of transcription in wheat. Immediately upon the draft genomes of wheat progenitors *T. urartu* and *Ae. tauschii* being available, they were used to study gene expression patterns during the production of newly synthesized hexaploid wheat. The RNA-seq and small RNA reads were aligned to a consensus genome produced from sequences common to the A and D draft genomes [29], [52] to avoid mapping bias during expression level estimation. Interesting results were obtained regarding the function of genes from different progenitors for growth vigor in hexaploid wheat [54]. The availability of the *Ae. tauschii* genome also accelerated the cloning of the *Male Sterile 2* (*MS2*) gene that is located on the short arm of the wheat chromosome 4D [55], [56]. Later on, the second fertility related genes were cloned [57], [58]. For genome-wide gene expression studies, both the IWGSC CSS and TGACv1 assemblies were used for transcript assembly to study molecular regulators in wheat spike development using a transcriptome association strategy for 90 wheat lines (74 landraces and 16 modern cultivars) [59]. The TGACv1 genome was also used to study genomic imprinting in diploid, tetraploid, and hexaploid wheat [60]. Further study of a total of 53,259 genes that were found in all three subgenomes, so called triads, showed only 30% of them with non-balanced expression, while a larger part of triads (72.5%) were expressed with similar levels, especially those located in syntenic regions [61]. Consistent with previous studies [62], no significant subgenome expression dominance was identified.

Genome sequences now allow comparison of divergence among wheat accessions. A number of versions of exon capture probes based on the early IWGSC CSS assembly have been designed and available for use. The 110-Mb NimbleGen (Roche) exome capture probes were the first generation and have been used to study the natural variation among 62 diverse wheat lines [63]. Selected regions associated with important agronomic traits have been identified by genome-wide association study (GWAS) and can now support wheat breeding. A more extensive study of a world-wide panel of about 500 genotypes were selected from a wide geographical range and studied by exome sequencing, revealing the consequence of 10,000 years of human selection and breeding on wheat adaptation and genetic shift [64]. Another large scale survey of 890 wild-relative introgression lines, by a similar exome sequencing approach, provided deeper insights into the adaptive landscape of the wheat genome and confirmed the role of historic gene flow from wild relatives to the adaptive diversity of modern wheat [65]. Genetic and methylation variations have also been studied among 104 landraces of common wheat using 12 Mb of the 110-Mb exon capture probes, revealing that SNPs may be preferentially “hard-coded” by 5-methylcytosine deamination of ancestral methylation states, a mechanism that could underline local adaption of wheat varieties [66]. Methylation studies using this approach showed conserved methylation patterns across the three subgenomes and between the D subgenome of hexaploid wheat and its progenitor *Ae. tauschii* [67]. Such an observation is exciting because accessions of *Ae. tauschii* have been widely used to transfer important genes for yield improvement and disease resistance through newly synthesized hexaploid wheat [68]. Toward this end, assisted by the D genome sequence, genome-wide DNA methylation in *Ae. tauschii* during the infection of obligate biotrophic fungus *Blumeria graminis* f. sp. *tritici*, which causes powdery mildew disease in both common wheat and *Ae. tauschii*, was studied. The work showed that DNA methylation status, especially methylation at CHH trinucleotides (H indicates any nucleotide other than C), was involved in fungal defense in *Ae. tauschii*, which may be conserved in common wheat [69].

## Perspectives

The *Three Kingdoms*-style competition for wheat genome sequencing reflects the desire of the wheat community for this pivotal information for gene discovery and breeding. The wheat reference genome sequence lends a strong hand to wheat researchers and breeders that have been available to other major crops for years. Now, the reliance on the synteny between wheat and model plants such as *Brachypodium distachyon* and rice is no longer essential [19], [70] although still valuable in functional annotation. Gene cloning will be significantly accelerated using strategies and methods such as bulk segregation analysis (BSA), MutMap, and bulk segregation of RNA-seq (BSR-seq) that have been routinely used in other species [57], [71], [72]. This has been exemplified by the cloning of the wheat *Fusarium head blight 1* gene [73], [74] that has been painstakingly investigated for more than 15 years without the reference genome of wheat [75]. Homoeolog specific primers can now be designed for gene expression analysis, single guide RNAs (sgRNAs) for CRISPR/Cas9 gene editing, and specific RNAi, without additional effort to obtain the genomic sequence. With the development of a comprehensively integrated database containing information for wheat whole genome genetic diversity, pangenome sequences, genetic mapping, and ideally breeding pedigrees, agronomically important traits can now be located more efficiently by GWAS. Together with more sophisticated databases [76], [77], the wheat genome sequences will thus increase our understanding of wheat biology through functional genomics studies, in parallel with breeders as they track genomic changes in their breeding schema to expedite the process.

Many long-awaited fundamental biological questions can now be addressed to define wheat evolution, domestication, polyploidization, as well as genetic and epigenetic interaction between homoeologous genes and genomes. Wheat will reestablish itself as a model for plant genetic research. As a young polyploid with a special mechanism to prevent genetic crossing-over between homoeologous chromosomes, wheat will be an ideal model to study polyploid genetics because homoeology has significantly been eroded in other (paleo-)polyploid plants such as maize, soybean, and cotton. With recent breakthroughs in wheat transformation [78], the advance of genome editing technologies [79] and speed breeding technologies [80], wheat can now catch up with its peer crops in genome-based molecular breeding. The spring of wheat research has finally arrived.

## Competing interests

The authors declare no competing interests.
